# Resolvin D1 inhibits the proliferation of osteoarthritis fibroblast-like synoviocytes through the Hippo-YAP signaling pathway

**DOI:** 10.1186/s12891-022-05095-1

**Published:** 2022-02-15

**Authors:** Siwei Su, Wenjun Jiang, Xiaoying Wang, Sen Du, Jianhong Qi, Qingwei Jia, Hongqiang Song

**Affiliations:** Shandong First Medical University (Shandong Academy of Medical Sciences), Tai’an, Shandong China

**Keywords:** Osteoarthritis, Resolvin D1, Hippo-YAP signaling pathway, Proliferation

## Abstract

**Objective:**

Osteoarthritis (OA) is a disease characterized by cartilage degradation and structural destruction. Resolvin D1 (RvD1), a specialized proresolving mediator (SPM) derived from omega-3 fatty acids, has been preliminarily proven to show anti-inflammatory and antiapoptotic effects in OA. However, the mechanisms of RvD1 in osteoarthritis fibroblast-like synoviocytes (OA-FLSs) need to be clarified.

**Methods:**

Synovial and fibroblast-like synoviocytes were obtained from OA patients and healthy individuals. MTT and EdU assays were performed to determine cell cytotoxicity and proliferation. The protein expression levels of cyclin D1, cyclin B1, PCNA, p53, MMP-13, YAP, p-YAP, and LATS1 were detected by western blot analysis. The release levels of IL-1β were detected by ELISA. The cell cycle was assessed by flow cytometry. Immunofluorescence was used to detect the levels of YAP in OA-FLSs.

**Results:**

RvD1 inhibited OA-FLS proliferation and reduced MMP-13 and IL-1β secretion in the concentrations of 20 nM and 200 nM. Furthermore, RvD1 induced G2 cell cycle arrest in OA-FLSs via the Hippo-YAP signaling pathway and promoted YAP phosphorylation. However, RvD1 had no effects on normal FLSs.

**Conclusions:**

RvD1 inhibits OA-FLS proliferation by promoting YAP phosphorylation and protects chondrocytes by inhibiting the secretion of MMP-13 and IL-1β, providing an experimental basis for RvD1 treatment of OA.

**Supplementary Information:**

The online version contains supplementary material available at 10.1186/s12891-022-05095-1.

## Introduction

Osteoarthritis (OA), a total joint disease, is the leading cause of disability and reduces the quality of life in elderly people [1]. The pathogenesis of OA involves changes in articular cartilage, subchondral bone, ligaments, joint capsule, synovium and surrounding muscle structure [2]. It is an active dynamic change caused by the imbalance between joint tissue repair and destruction [3].

Histological changes occurring in the osteoarthritis synovial membrane include macrophage infiltration, synovial lining hyperplasia, and the production of inflammatory cytokines [4]. However, hyperplasia of synovial tissue can lead to the synthesis and release of numerous cytokines, chemokines, matrix metalloproteinases, and collagenase, leading to more severe synovitis; therefore, inhibition of osteoarthritis fibroblast-like synoviocyte (FLS) inflammation and hyperproliferation will contribute to the treatment of OA [5, 6].

The Hippo signaling pathway controls organ size, plays a key role in the regulation of tissue homeostasis and regulates cell number by modulating cell proliferation and cell death [7]. At the center of the pathway is a kinase cascade composed of MST1/2, SAV, LATS1/2, and MOB1A/B [8]. When the kinase cascade module is activated, a series of phosphorylated proteins activate the downstream kinases LATS1/2, which in turn phosphorylate YAP/TAZ, the key effectors of the pathway, leading to the cytoplasmic sequestration or proteasomal degradation of YAP/TAZ [9]. Conversely, inactivation of the Hippo pathway increases YAP/TAZ nuclear translocation, thereby activating TEAD transcription factors to promote downstream gene transcription [9]. Deng et al. [10] Previous studies have shown that YAP plays an indispensable role in the maintenance of cartilage homeostasis in osteoarthritis. Bottini et al. [11] showed that YAP promoted TGF-β-dependent SMAD3 nuclear localization in rheumatoid arthritis FLSs and that inhibition of YAP alleviated rheumatoid arthritis. However, the mechanism of the Hippo-YAP signaling pathway in OA-FLSs is still unclear.

Resolvin D1 (RvD1), a specialized pro-resolving mediator (SPM) derived from omega-3 fatty acids, has been reported to play an unexpected therapeutic role in many diseases, especially as an endogenous and strengthening anti-inflammatory mediator [12]. Houda et al. [13] found that RvD1 can modulate inflammatory and catabolic responses in OA chondrocytes. Antonia et al. [14] discovered that RvD1 ameliorates obesity-induced OA by decreasing macrophage infiltration in the synovium and regulating synovial macrophage polarization. Recently, Sun et al. [15] found that RvD1 plays a suppressive role in pannus formation by upregulating miRNA-146a-5p in rheumatoid arthritis. However, the molecular mechanism by which RvD1 alleviates OA and its relationship with the Hippo-YAP signaling pathway are still unknown.

In this study, we aimed to discover whether RvD1 alleviates OA via the Hippo-YAP signaling pathway and illustration the function of RvD1 in OA.

## Material and methods

### Patients and samples

Human synovium was obtained from OA patients (*n* = 4) who underwent artificial total knee arthroplasty at the Second Affiliated Hospital of Shandong First Medical University. Patients had advanced disease and were diagnosed with primary OA, excluding trauma and other structural causes of secondary OA. Control samples from healthy individuals (*n* = 3) were obtained from patients at the time of knee arthroscopic evaluation. These patients were diagnosed as a result of knee injury caused by trauma to the knee or sport injury, excluding rheumatic diseases and inflammation. This study was approved by the Clinical Research Ethics Committees of the Second Affiliated Hospital of Shandong First Medical University. All patients participating in this study signed informed consent forms.

### Cell culture

FLSs were obtained via enzyme digestion and tissue cultivation. In short, after rinsing with phosphate-buffered saline (PBS), synovial tissues were sliced into 1 mm^3^ sections using sterile ophthalmic scissors and put into 15 ml centrifuge tubes. Next, 1 mg/ml collagen II enzyme (Solarbio, China) was added to the tube, which was four times larger than the tissues. After thorough mixing, the samples were incubated with 5% CO_2_ in air at 37 °C for 2 h. Then, the tissue digestion fluid was filtered with stainless steel meshes (200) and centrifuged at a rate of 1500 r/min for 5 min. After discarding the supernatant, the samples were cultured in Dulbecco’s modified Eagle’s medium (DMEM) supplemented with 12% fetal bovine serum (FBS), 100 U/ml penicillin, and 100 μg/ml streptomycin in a 37 °C, 5% CO_2_ incubator. The undigested tissue pieces were continuously cultured. All experiments were performed between passage 3 and 5 of cell culture.

### MTT assay

Cells were seeded in 96-well plates at a density of 5 × 10^3^ cells/well. After overnight incubation, OA-FLSs were treated with different concentrations (0, 20, 50, 100 and 200 nM) of RvD1 (Cayman Chemical Company, USA) for 48 h. Subsequently, 20 μL MTT solution (0.5 mg/mL) was added to each well, and the plates were incubated for 4 h in a 5% CO2 atmosphere at 37 °C. After a brief centrifugation, the culture medium was removed, and 150 μL (Dimethyl sulfoxide) DMSO was added to each well. The absorbance at 490 nm was measured with a spectrophotometer.

### Western blot analysis

Tissue and Cultured cells were collected and lysed in RIPA buffer (Servicebio, China) supplemented with 100 x PMSF (Servicebio, China) and 100 x phosphatase inhibitor (Servicebio, China). The protein concentrations were determined with a BCA protein assay kit (Solarbio, China), and adjust the final protein concentration to 2 μg/μL. The proteins were separated by SDS–PAGE and transferred onto polyvinylidene fluoride (PVDF) membranes. The membranes were blocked with TBST containing 5% bovine serum albumin for 1 h and then incubated with the antibody overnight at 4 °C. The antibodies used for this assay were cyclin D1 (Santa Cruz, USA, 1:1000), cyclin B1 (Abcam, UK, 1:1000), PCNA (Santa Cruz, USA, 1:1000), p53 (Santa Cruz, USA, 1:1000), MMP-13 (Santa Cruz, USA, 1:1000), YAP (CST, USA, 1:1000), p-YAP (CST, USA, 1:1000), and LATS1 (CST, USA, 1:1000). After washing with TBST, the blots were incubated with HRP-labeled secondary antibodies (Servicebio, China, 1:5000) for 2 h at room temperature. The blots were visualized by using an enhanced chemiluminescence reagent kit.

### Immunofluorescence

FLSs were washed in PBS, fixed in 4% paraformaldehyde and treated in 0.5% Triton X-100 for 10 min. After rinsing, the cells were blocked with 5% bovine albumin for 1 h at ambient temperature, rinsed with PBS and incubated with primary antibody for YAP (1:100), diluted in 3% BSA overnight at 4 °C. After washing and incubation with secondary antibodies for 1 h at ambient temperature, the cells were labeled with DAPI for 5 min. A fluorescence microscope (Nikon) was used for observation.

### Elisa

The IL-1β in FLS culture filtrate cytokines were detected using specific ELISA kits (Beyotime Biotechnology, China), according to the manufacturer’s instructions.

### Cytometry analysis

Cells were collected and washed twice with ice-cold PBS and then fixed with 75% ethanol at 4 °C for 2 h. After washing 2 times with ice-cold PBS, cells were incubated with 100 μl RNase A for 30 min at 37 °C. Then, (Propidium Iodide) PI (Solarbio, China) was added to each of the tubes to achieve a final concentration of 50 μg/mL, and the tubes were incubated in the dark at 4 °C for 30 min. The samples were then analyzed by flow cytometry.

### EdU labeling and analysis

The 5-ethynyl-2-deoxyuridine (EdU) incorporation assay was used to detect the proliferation of FLSs. FLSs were seeded in 96-well plates at a density of 5 × 10^3^ cells/well. After night, RA-FLSs were treated with RvD1 for 48 h. EdU was added to each well and cultured for another 3 h. The KFluor488-EdU cell proliferation detection kit (Beyotime Biotechnology, China) was used to assess proliferation following the manufacturer’s instructions.

### Statistical analysis

The data are presented as the mean ± standard deviation (SD). Statistical analysis was performed by Student’s t-tset for two groups or one-way analysis of variance (ANOVA) or two-way ANOVA with repeated measures. All experiments were performed at least three independent times. NS, not significant, *P < 0.05; ** *P* < 0.01. GraphPad Prism 8.0 were used to analyze the data.

## Results

### Identification of human FLSs

**Fig. 1:**
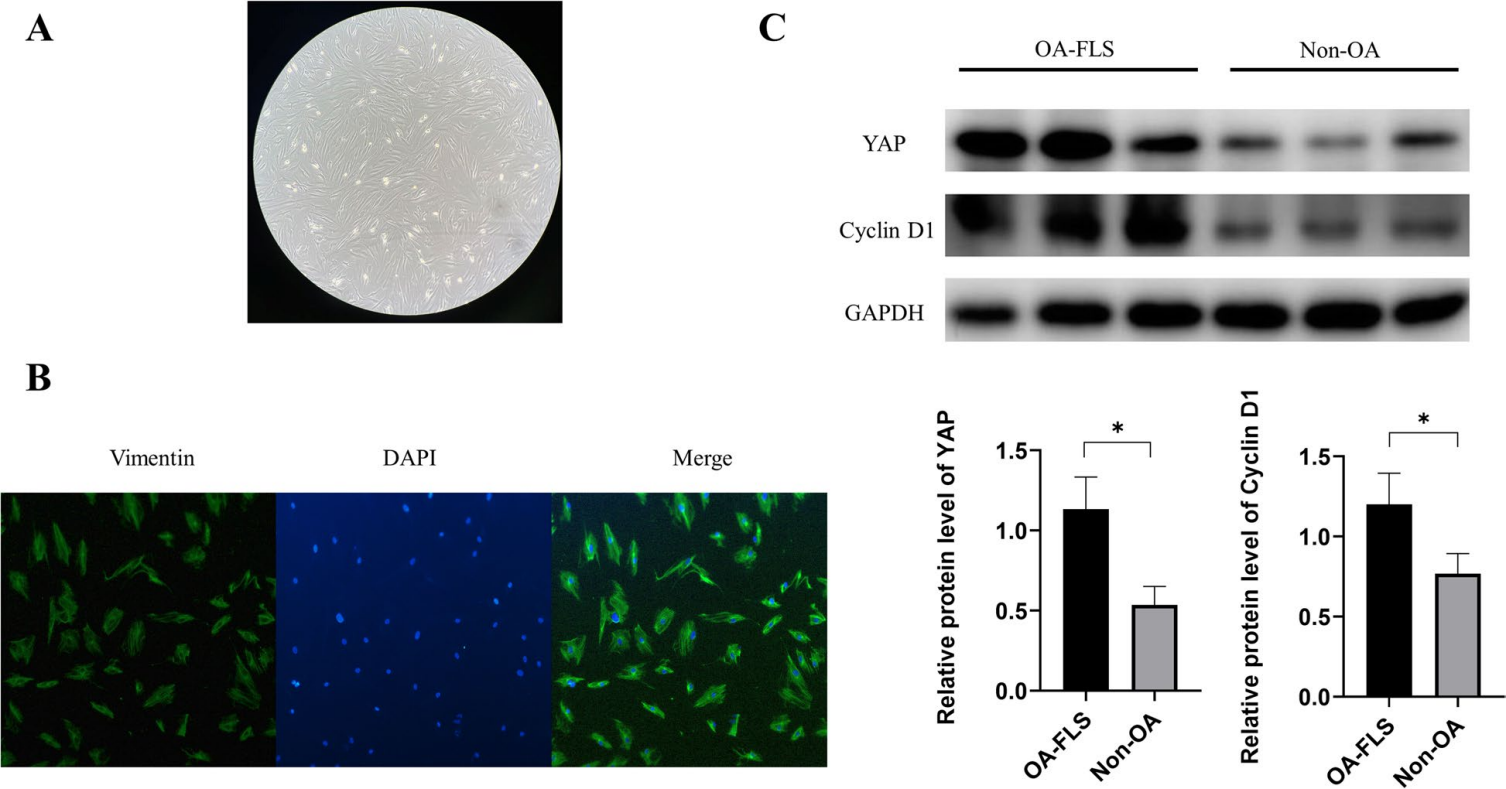
FLS extraction and identification and synovial tissue detection. **A** Optical microscopy showed third generations of FLS fusiform (100× magnification). **B** Immunofluorescence staining of vimentin in FLSs (200 × magnification). **C** YAP and cyclin D1 expression was measured by western blot analysis in normal and OA synovium. *P < 0.05 as comparison with OA-FLS group. All experiments were repeated three times independently

First, we identified FLSs obtained from human synovial tissues through morphology and immunofluorescence. After cultivation for 4 days, a mass of cells around the tissue gradually climbed out. After one week of cultivation, the cells covered approximately 80% percent of the bottom of the petri dish and grew radially (Fig. 1A). When observed under an optical microscope, the cells were long fusiform and relatively large in volume. Vimentin immunostaining were performed on FLSs. The results showed that vimentin was positive (Fig. 1B), proving that the cells obtained were FLSs.

### Protein expression of YAP and cyclin D1 in non-OA and OA patient synovial tissues

To explore the effect of YAP and cyclin D1, we first examined the protein expression of YAP and cyclinD1. As shown in Fig. 1C, both YAP and cyclin D1 were observably upregulated in synovial tissues of OA patients compared with non-OA synovial tissues.

### RvD1 has no cytotoxicity in normal human FLSs

**Fig. 2:**
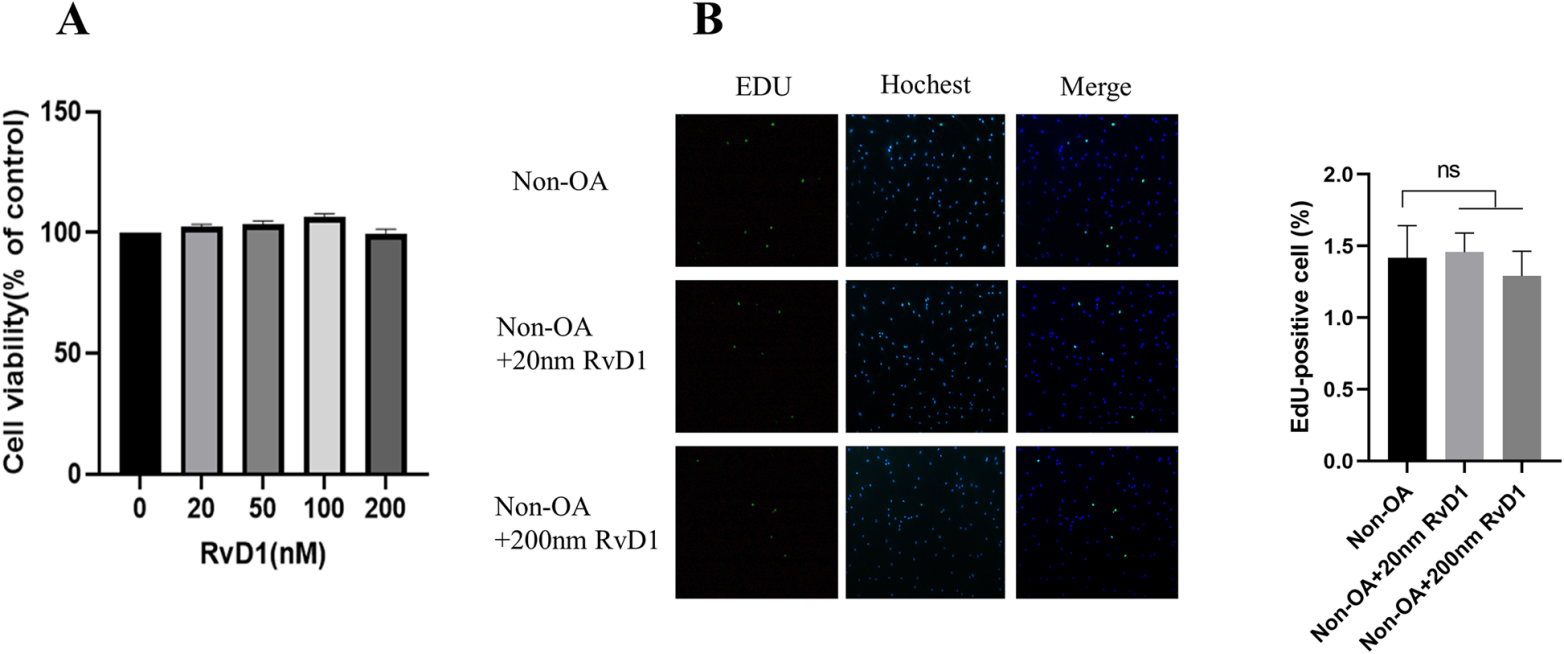
RvD1 have no effect on normal FLSs. **A** Normal FLSs were incubated with RvD1 (0, 20, 50, 100 or 200 nM), and cell viability was detected by MTT assay. **B** Normal FLSs were incubated with RvD1 (0, 20 or 200 nM), and cell proliferation was detected by EdU assay (100 × magnification). *P < 0.05 as comparison with OA-FLS group. All experiments were repeated three times independently

This part of the experiment was to test the cytotoxic effect of RvD1 on human FLSs. As shown in Fig. 2A, RvD1 at different concentrations (0–200 nM) had no cytotoxicity in cells after 48 h of incubation. To further explore the effect of RvD1 on the proliferation of FLSs, an EdU assay was performed on non-OA-FLSs treated with different concentrations of RvD1 (0 nM, 20 nM, and 200 nM) for 48 h. We found that RvD1 had no effect on the proliferation of non-OA-FLSs (Fig. 2B).

### RvD1 inhibits the proliferation of OA-FLSs by arresting the cell cycle

**Fig. 3:**
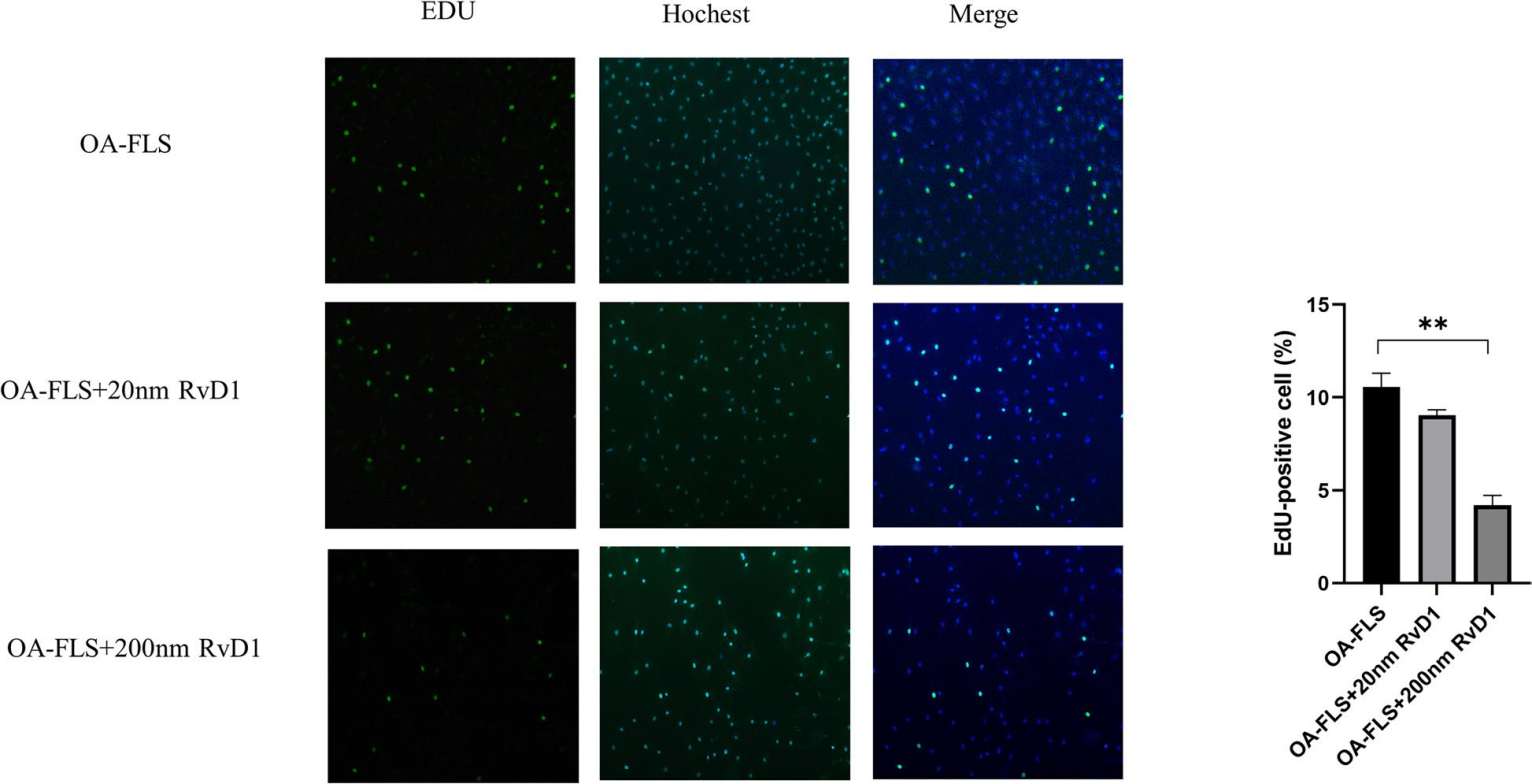
RvD1 inhibit the proliferation of OA-FLSs. OA-FLSs were incubated with RvD1 (0, 20, and 200 nM), and cell proliferation was detected by EdU assay (100 × magnification). **P < 0.01 as comparison with OA-FLS group. All experiments were repeated three times independently

The control of proliferation of the synovial membrane in OA patients has a protective effect on cartilage [16]. To investigate the effect of RvD1 on the proliferation of OA-FLSs, an EdU assay was performed on OA-FLSs incubated with different concentrations of RvD1 (0 nM, 20 nM, and 200 nM) for 48 h. The results showed that RvD1 inhibited OA-FLS growth in the concentrations of 20 nM and 200 nM (Fig. 3). To further investigate the possible mechanism of restraining the proliferation of OA-FLSs by RvD1, we studied the effect of RvD1 on the cell cycle. After incubation with different concentrations of RvD1 (0 nM, 20 nM, and 200 nM) for 48 h, OA-FLSs were detected by flow cytometry.

**Fig. 4:**
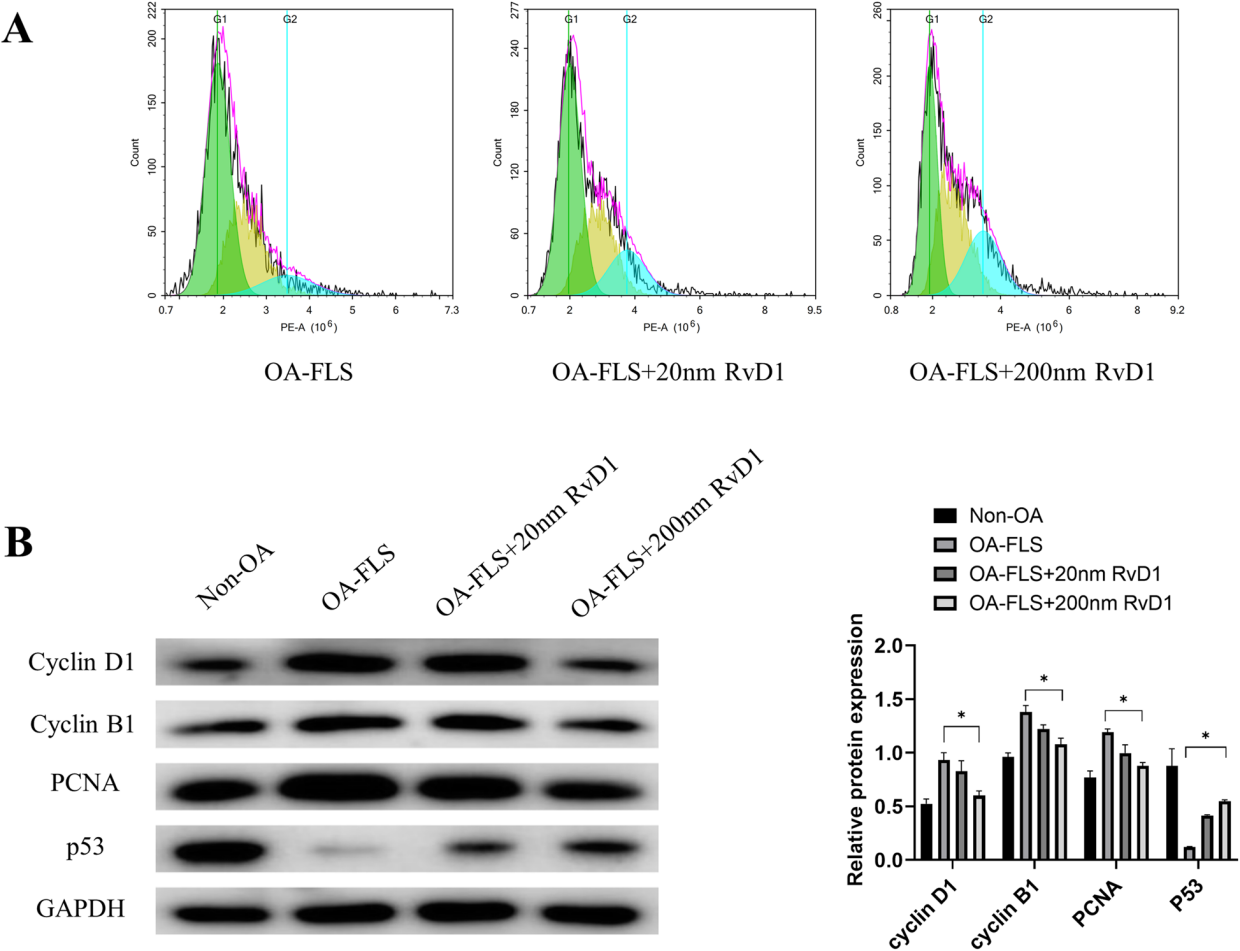
RvD1 regulates OA-FLSs cell cycle progression. **A** The cell cycle of OA-FLSs incubated with RvD1 (0, 20, and 200 nM) for 48 h was detected using PI staining followed by flow cytometry. **B** Cyclin D1, cyclin B1, PCNA, and p53 were measured by western blot analysis in non-OA FLSs and OA-FLSs after incubation with RvD1 (0, 20, and 200 nM) for 48 h. *P < 0.05 as comparison with OA-FLS group. All experiments were repeated three times independently

The results showed that RvD1 may restrain cell proliferation by causing G2 phase cell arrest (Fig. 4A). To further understand how RvD1 arrests the cell cycle, we examined the protein expression of cell proliferation and cell cycle proteins. Western blot results showed that the protein levels of cyclin D1, cyclin B1, and PCNA were downregulated, while the protein levels of p53 were upregulated in OA-FLSs after incubation with RvD1 for 48 h (Fig. 4B).

### RvD1 suppresses the expression of MMP13 and IL-1β in OA-FLSs

**Fig. 5:**
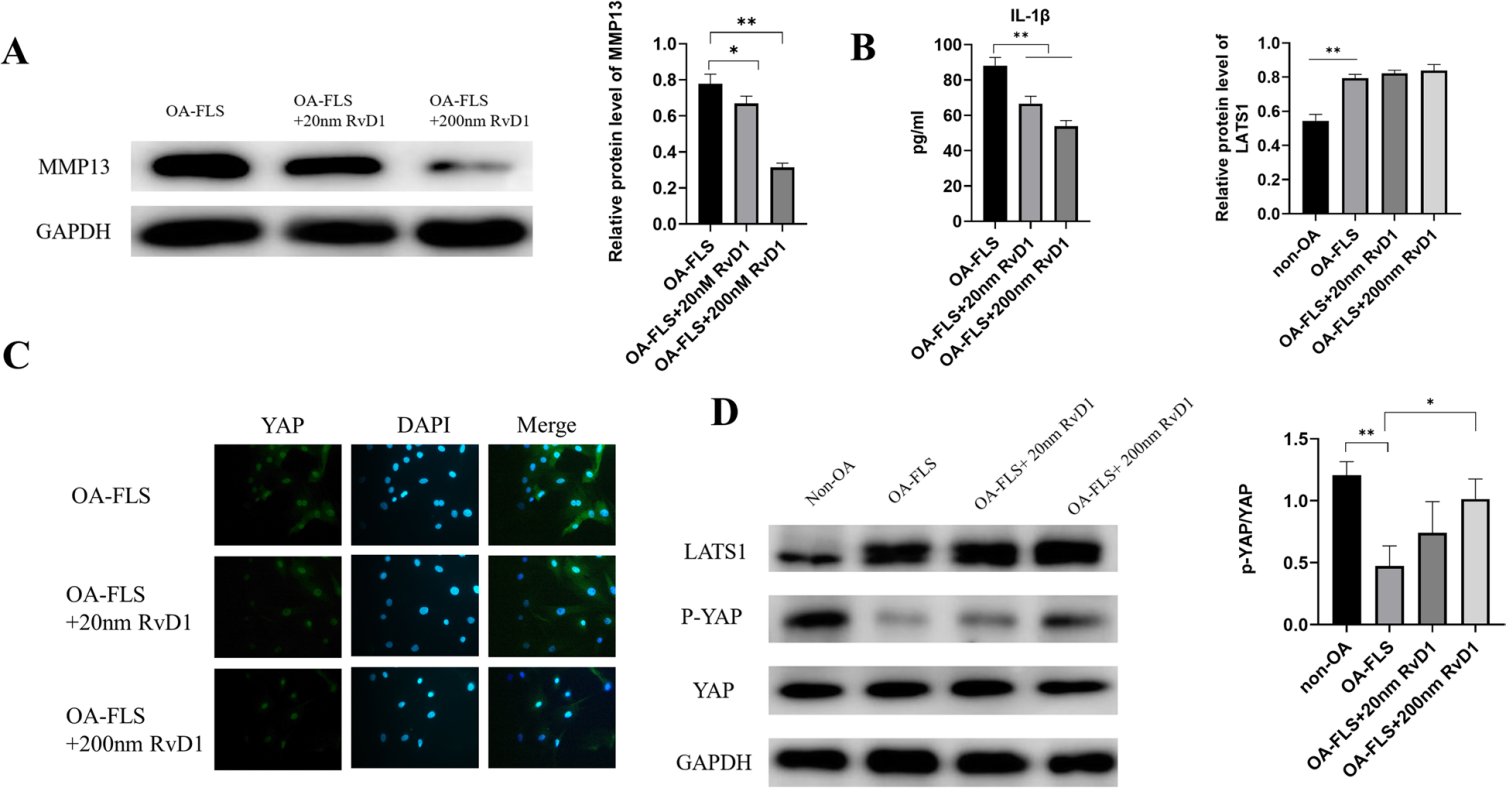
RvD1 inhibits the expression of MMP-13, and IL-1β and promotes YAP phosphorylation. **A** MMP-13 was measured by western blot analysis in OA-FLSs after incubation with RvD1 (0, 20, and 200 nM) for 48 h. **B** IL-1β was measured by ELISA in OA-FLSs after incubation with RvD1 (0, 20, and 200 nM) for 48 h. **C** YAP was detected by immunofluorescence staining in OA-FLSs after incubation with RvD1 (0, 20, and 200 nM) for 48 h (400 × magnification). **D** LATS1, p-YAP, and YAP were measured by western blot analysis in OA-FLSs after incubation with RvD1 (0, 20, and 200 nM) for 48 h. *P < 0.05; **P < 0.01 as comparison with OA-FLS group. All experiments were repeated three times independently

Research has proven that the high expression of inflammatory cytokines and matrix metalloproteinases may aggravate the progression of OA [17]. The expression of IL-1β and MMP13 in OA-FLSs incubated with RvD1 was examined by ELISA and western blot separately. As shown in Fig. 5A, B, the expression of IL-1β and MMP13 was significantly downregulated by RvD1. These data indicated that RvD1 could decrease the inflammatory response in OA-FLSs.

### RvD1 promotes the phosphorylation of YAP in OA-FLSs

It has been reported that YAP inhibition reduces FLS pathogenesis in RA. Therefore, we evaluated the expression of YAP in OA-FLSs under RvD1 treatment. Immunofluorescence results showed that YAP expression was downregulated in OA-FLSs incubated with different concentrations of RvD1 (0 nM, 20 nM, and 200 nM) for 48 h (Fig. 5C). Studies have shown that phosphorylation leads to the cytoplasmic sequestration of YAP, which in turn causes proteasomal degradation. The western blot results showed that the expression of p-YAP was upregulated in OA-FLSs incubated with different concentrations of RvD1 (0 nM, 20 nM, and 200 nM). In contrast, the high expression of LATS1 was not reversed under RvD1 treatment (Fig. 5D). These data implied that RvD1 downregulated the expression of YAP by increasing the phosphorylation of YAP in OA-FLSs.

### Effects of RvD1 and verteporfin on OA-FLSs

**Fig. 6:**
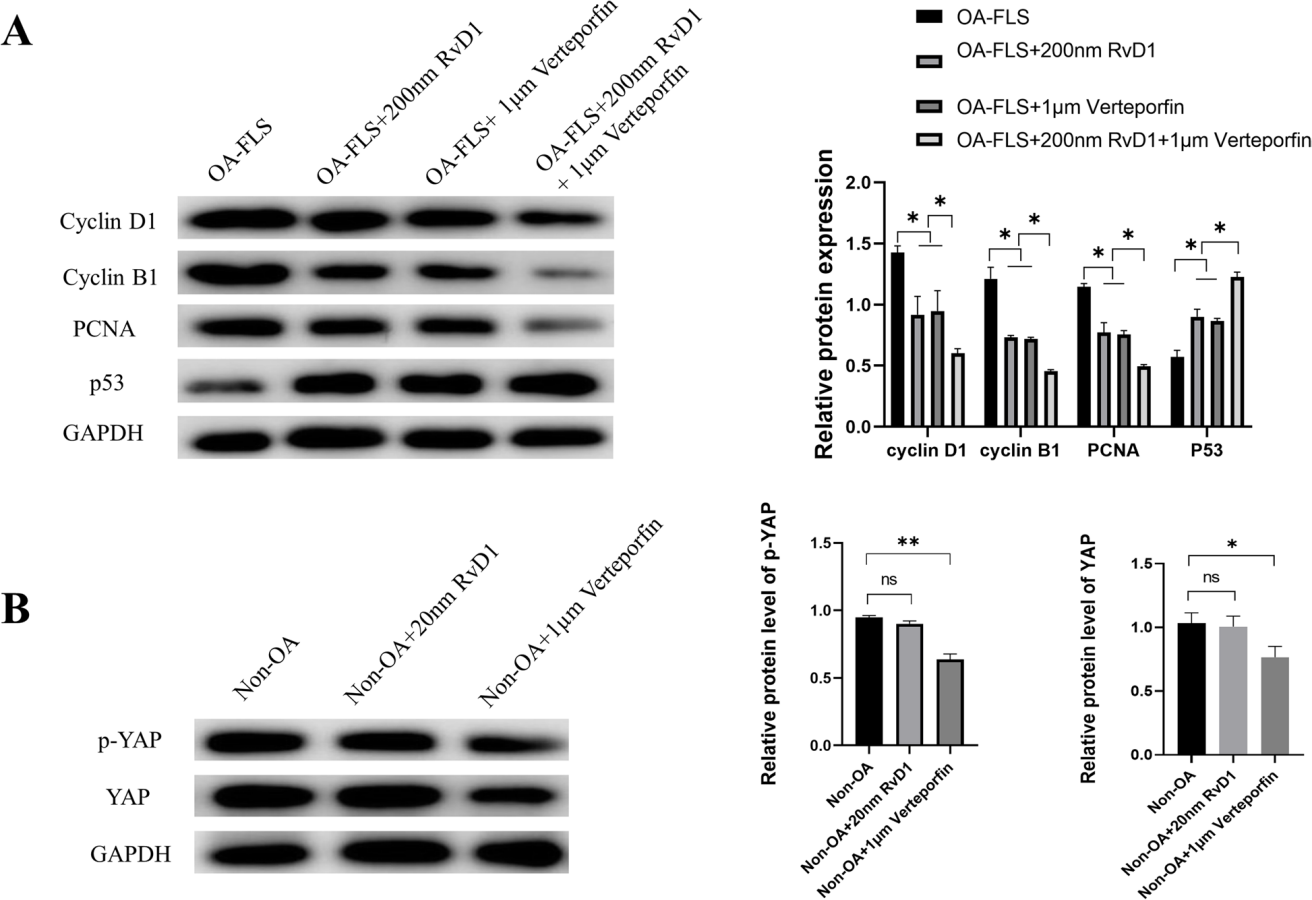
RvD1 regulates the OA-FLS cell cycle by regulating YAP expression. **A** Cyclin D1, cyclin B1, PCNA, and p53 were measured by western blot analysis in OA-FLSs after incubation with 200 nM RvD1 or 1 μM verteporfin or both RvD1 and Verteporfin for 48 h. **B** YAP was measured by western blot analysis in non-OA FLSs after incubation with 200 nM RvD1 or 1 μM verteporfin for 48 h. ns: not significant; *P < 0.05; **P < 0.01 compared with the OA-FLS group. All experiments were repeated three times independently

We treated OA-FLSs with the small molecule verteporfin, a YAP inhibitor that disrupts YAP-TEAD interactions in vitro and in vivo. Consistent with the observed effect of RvD1, inhibition of YAP with 1 μM verteporfin for 48 h in OA-FLSs downregulated the expressions of cyclin D1, cyclin B1, and PCNA, while the expression of p53 was upregulated. In addition, the expressions of cyclin D1, cyclin B1, and PCNA were downregulated in OA-FLSs group after 48 h of both RvD1 and verteporfin treatment but p53 was upregulated (Fig. 6). However, verteporfin also inhibited the expressions of YAP and p-YAP in non-OA-FLSs; in contrast, the expressions of YAP and p-YAP in non-OA-FLSs incubated with RvD1 was not changed. Collectively, these results implied that RvD1 has no biological effect on the normal synovial membrane compared with verteporfin.

## Discussion

OA, the most common form of arthritis in adults, is a disease characterized by cartilage degradation and structural destruction. The histological changes that occur in the OA synovium include hypertrophy and hyperplasia with an increase in the number of synovial lining cells [18]. Multiple studies have indicated the involvement of OA-FLSs in the process of cartilage degradation through the production of catabolic and proinflammatory mediators such as cytokines and matrix metalloproteinases. In addition, hypertrophy of the synovial membrane aggravates this process [19]. RvD1, an endogenous lipid medium derived from ω-3, has been demonstrated to have anti-inflammatory and anti-apoptosis effects and promote tissue repair and is considered to be effective for a range of disorders, such as liver ischemia and reperfusion, arthritis, and non-small cell lung cancer [20–23]. For OA, RvD1 was identified as an antiapoptotic, anti-inflammatory, and antioxidant compound through multiple mechanisms [13]; however, the mechanisms and therapeutic effect of RvD1 on OA-FLSs are still ambiguous.

First, our EdU assay results showed that RvD1 can significantly inhibit the proliferation of OA-FLSs in the concentrations of 20 nM and 200 nM, proving that RvD1 exerts an antiproliferative effect on OA-FLSs. In addition, we investigated whether RvD1 has a side effect on normal FLSs. The MTT and EdU assay results showed that RvD1 was not cytotoxic in normal FLSs and had no effect on proliferation in normal FLSs. RvD1 inhibits the proliferation of various types of cancer cells through different pathways, including apoptosis or the cell cycle. To investigate the antiproliferative effect of RvD1 on OA-FLSs, we performed flow cytometry to examine the cell cycle of OA-FLSs. As a result, we observed significant G2 phase arrest in OA-FLSs after incubation with RvD1 for 48 h. As a checkpoint, G2/M plays a vital role in the normal cell cycle by preventing uncopied and DNA-damaged cells from entering mitosis before repairing it [24, 25]. To investigate the mechanism by which RvD1 regulated OA-FLS proliferation and the cell cycle, we detected the expression of proliferation- and cell cycle-related proteins. Western blot analysis indicated that the expression levels of cyclin D1 and cyclin B1 as well as PCNA were downregulated, while the protein levels of p53 were upregulated by incubation with RvD1. PCNA is a marker of cell proliferation, cyclin D1 and cyclin B1 are cell cycle-promoting proteins, and p53 is a growth suppressor protein. This result was in accord with the results examined in human synovial tissues, which showed that the protein expression of cyclin D1 was upregulated in OA-FLSs compared with non-OA FLSs (Fig. 1C). These results supported that RvD1 could inhibit the proliferation and arrest the cell cycle of OA-FLSs. As a proinflammatory cytokine, the secretion of IL-1β can trigger inflammation in chondrocytes and synovium; moreover, IL-1β affects MMP synthesis by chondrocytes, including MMP-1 and MMP13, which, in turn, destroy articular cartilage [26]. Therefore, inhibiting the secretion of IL-1β and MMPs is an effective way to prevent the progression of OA [27]. In this study, after incubation with RvD1, we observed that the expression of IL-1β and MMP13 was downregulated. Research has shown that activated synovial cells in the inflamed synovium produce catabolic and proinflammatory mediators, and the progression of OA and the activation of synovial cells are virtually interrelated [28]. Therefore, the downregulation of IL-1β and MMP-13 may contribute to the inactivation of OA-FLSs caused by RvD1 therapy.

The Hippo signaling pathway has been shown to play a central role in the regulation of organ size, regulation of migration and invasion, maintenance of cell proliferation and apoptosis balance, and YAP is the core protein in this pathway [29]. Wu et al. [30] showed that overexpression of YAP promotes cell proliferation and invasion in osteosarcoma, and downregulation of SPRX2 increased YAP phosphorylation, leading to reduced nuclear translocation to activate the Hippo signaling pathway. Bottini et al. [11] showed that YAP promotes RA FLS invasiveness in vivo and arthritis severity in mice, and inhibition of YAP with verteporfin was shown to reduce FLS pathogenesis in RA. However, no studies have been conducted to investigate the expression of YAP in OA synovial tissue. The present study demonstrated that the protein expression of YAP was upregulated in human OA synovial tissue compared with normal synovial tissue (Fig. 1C). Immunofluorescence assay results showed that the expression of YAP was downregulated after incubation with RvD1 (0 nM, 20 nM, and 200 nM). Research has indicated that the state of activation of the Hippo pathway eventually results in the phosphorylation of YAP and promotes its proteasomal degradation [9]. Therefore, we next investigated the level of phosphorylation of YAP. Experimental results show that the level of phosphorylation of YAP was elevated after incubation with RvD1 (0 nM, 20 nM, and 200 nM). In addition, the results demonstrated that the protein expression of LATS1/2 was upregulated in OA-FLSs compared with the normal group; importantly, treatment with RvD1 in OA-FLSs did not downregulate the expression of LATS1/2. These results implied that RvD1 possibly plays a role by regulating YAP phosphorylation in OA-FLSs. Verteporfin, as an inhibitor of YAP, acted as corroborative evidence of RvD1 therapy. We investigated whether RvD1 has a similar effect to verteporfin in inhibiting the expression of YAP and exerting antiproliferative effects. Nevertheless, in normal synoviocytes, verteporfin treatment decreased YAP expression, while RvD1 did not affect normal YAP expression. These results implied the targeted therapeutic effect of RvD1 in the pathological synovial membrane. The shortage of our study was it did not verify the therapeutic effect and mechanism of RvD1 in vivo. Therefore, in vivo experiments are pending for further study.

## Conclusion

In conclusion, YAP plays a key role in the pathogenesis of OA-FLSs. RvD1 alleviated overproliferation of OA-FLSs by promoting YAP phosphorylation, thereby attenuating the progression of OA, providing an experimental basis for RvD1 treatment of OA.

## Supplementary Information

**Additional file 1.**

## Data Availability

The datasets used or analyzed during the current study are available from the corresponding author on reasonable request.

## Abbreviations

OA: Osteoarthritis
RvD1: Resolvin D1
SPMs: Specialized pro-resolving mediator
OA-FLS: Osteoarthritis fibroblast-like synoviocyte
YAP: Yes-associated protein 1
