# Supplementary figures and images for: Resolvin D1 inhibits the proliferation of osteoarthritis fibroblast-like synoviocytes through the Hippo-YAP signaling pathway
Source: BMC Musculoskelet Disord. 2022 Feb 15;23:149. doi: 10.1186/s12891-022-05095-1 (PMC8845241; doi:10.1186/s12891-022-05095-1)

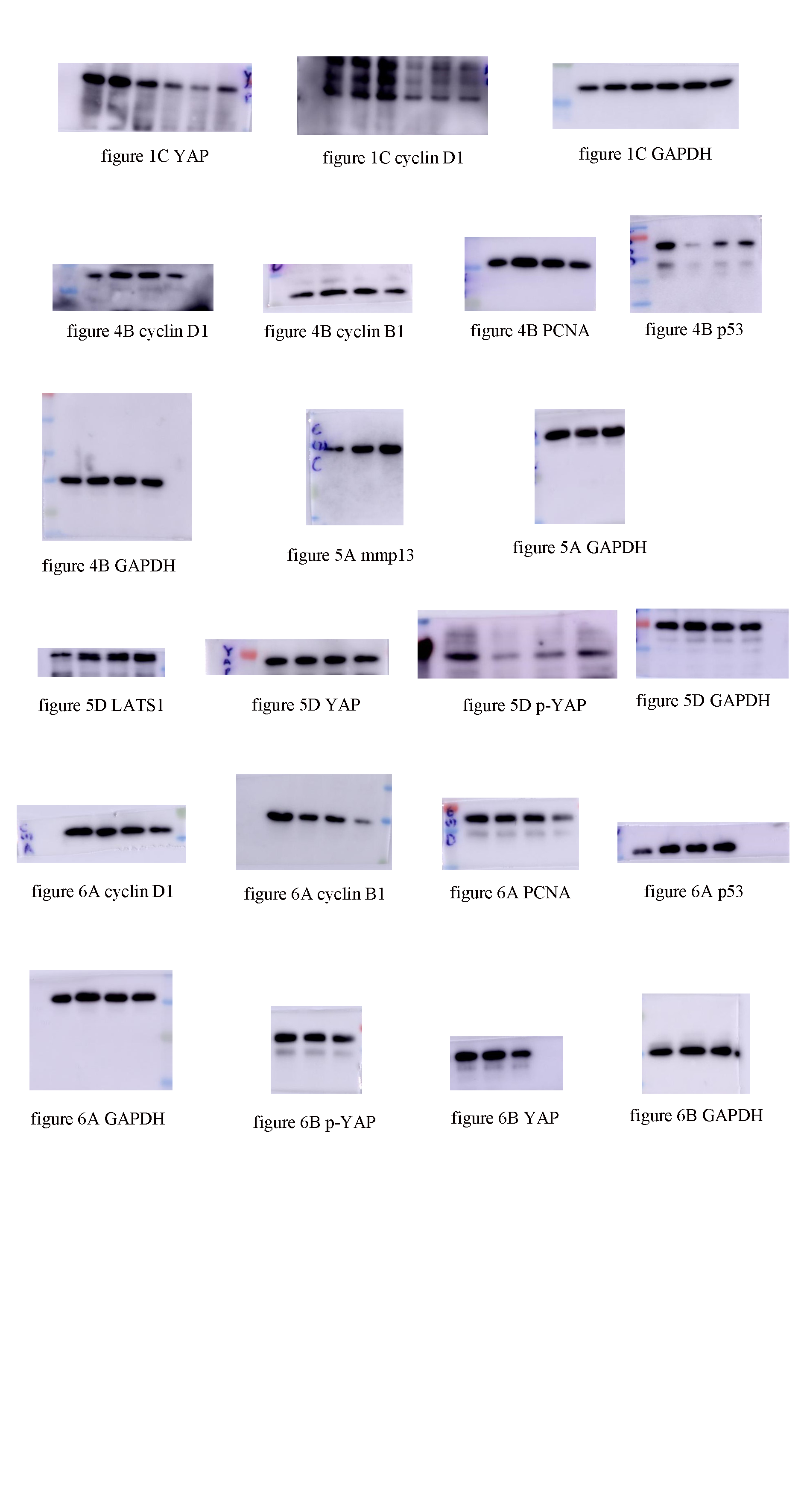

Supplement: Supplementary file 1 — Additional file 1. [file 12891_2022_5095_MOESM1_ESM.tif]
